# Development of Photocurable Polyacrylate-Based PolyHIPEs and the Study of the Kinetics of Photopolymerization, and of Their Thermal, Mechanical and Hydrocarbon Absorption Properties

**DOI:** 10.3390/polym13203497

**Published:** 2021-10-12

**Authors:** Ricardo Acosta Ortiz, Jefferson Alberto Reinoza Dávila, Ramiro Guerrero Santos

**Affiliations:** Centro de Investigación en Química Aplicada, Blvd Enrique Reyna No. 140, Saltillo 25294, Mexico; reinozaja.d17@ciqa.edu.mx (J.A.R.D.); ramiro.guerrero@ciqa.edu.mx (R.G.S.)

**Keywords:** acrylate, hydrocarbon absorption properties, high internal phase emulsion, HIPE, photopolymerization, kinetics, morphology

## Abstract

This article describes a comprehensive study to obtain polymeric porous materials via a photopolymerization technique, using acrylate-based high internal phase emulsions (HIPEs), as a template. The aim of obtaining these polymers was to use them as hydrocarbon absorbing materials. Kinetics of photopolymerization of the acrylate monomers and of the HIPEs were conducted to optimize the process. The obtained monoliths were characterized by thermal analysis such as differential scanning calorimetry (DSC) and thermogravimetric analysis (TGA). The morphology and surface area were analyzed by scanning electron microscopy (SEM) and Brunauer–Emmett–Teller (BET) analysis. The compression properties of the materials were determined, as well as their absorption properties of hydrocarbons such as hexane, diesel, toluene and chloroform. The findings show that the acrylate-HIPEs displayed high reactivity photopolymerizing in 20 min. The glass transition temperature of the materials were in the range of 2 to 83 °C, depending on the ratio of acrylates in the photocurable formulation, displaying the characteristic morphology with voids and interconnecting windows. The polyHIPEs exhibited superior properties of absorption of the studied hydrocarbons. The order of capability of absorption was chloroform > toluene > hexane > diesel. The optimum absorbing material was that with trimethylolpropane triacrylate, ethylhexyl acrylate and isobornyl acrylate in a 1:0.9:2.1 ratio, which absorbed 778% of chloroform, 378% of toluene, 306 % of hexane and 236% of diesel.

## 1. Introduction

Synthesis of porous polymers is well-researched and part of the historical development of porous materials that is a mainstream trend in both scientific and technological fields [1]. Pertinent definitions and the evolution of the research set forth below demonstrate that these materials display advantages such as high specific surface area, controlled size of pores, ease of functionalization and numerous applications [2]. There are a diversity of methods to obtain porous materials with well-defined porosity [3,4,5].

Emulsion-templated polymers derived from high internal phase emulsions (HIPEs), which are generally known as polyHIPEs, are a special type of porous materials [6,7,8]. The HIPEs are also known as highly concentrated emulsions, high internal phase ratio emulsions (HIPREs), gel emulsions, or hydrocarbon gels. These emulsions are biphasic systems composed of at least 74% of the internal phase volume and usually stabilized by a surfactant soluble in the continuous phase [9,10]. The polyHIPEs can be obtained with different shapes like monoliths, films, beads or rods, displaying closed or open interconnected pores that allow the exchange or mobility of substances through the material [11,12,13,14,15,16,17,18].

Conventional free radical polymerization (FRP) is the most reported synthesis method in the literature for polyHIPEs preparation. This method requires that the components of the oleic phase (e.g., monomers, surfactant, etc.) allow an adequate propagation of radicals in the medium, through the polymerizable bonds. Furthermore, the emulsion should be stable enough to maintain its characteristic polyhedral structure. There are different initiating mechanisms reported for the HIPEs polymerization, e.g., via redox generation of radicals [19], thermal decomposition of azo or peroxy initiators [20] and photoinitiation [21]. The thermal initiation is problematic since the HIPE stability is highly compromised. Photoinitiation does not require thermal activation, therefore this initiation method should offer important advantages in terms of stability and ease to generate polyHIPEs. However, the usage of photoinitiation is relatively unemployed and scarcely studied to date.

Reports about polyHIPEs synthesis began in the 1980s decade [21,22], however the first porous monolith synthesized by HIPEs photocuring (also known as photo-polyHIPEs) was reported in 2006 [23]. Since that year, a series of investigations about photo-polyHIPEs synthesis from formulations composed of different monomers, surfactants, photoinitiators and aqueous phases (mostly as internal phase) have been reported [18,24,25,26,27,28,29,30,31,32,33,34,35]. The obtained materials from these investigations have shown good performance in medical applications and biochemical processes [23,28,36,37,38,39,40,41], filtration, separation and chromatography processes [42,43,44,45,46], electrical energy production/storage, [47,48,49], support for catalysts [50] and as absorbent materials for the removal of hazardous materials [51].

However, there are two specific research gaps that have not been studied or developed in-depth: (1) the photocuring rate of the monomers in the HIPE, and its difference with respect to the polymerization rate of pristine monomers, and (2) the use of photo-polyHIPEs as hydrocarbon selective sorbents. Therefore, this research focuses on identifying the effect of different parameters, like the composition of the HIPE, the reactivity of the involved monomers, the type and concentration of photoinitiators and the intensity of radiation source, on the stability of the HIPE and on the morphology and mechanical properties of the porous polymeric material. Additionally, the capability of polyHIPEs to absorb hydrocarbon organic solvents by controlling the morphology, is a matter of practical application.

The extent of existing literature and the gaps in research are foundational to the aim of this research to obtain porous materials that could act as sorbent of solvents, using a rapid and efficient method of photopolymerization. Acrylate monomers like ethylhexyl acrylate, trimethylolpropane triacrylate and isobornyl acrylate were evaluated to obtain the porous monoliths. Kinetics of photopolymerization were determined in both, pristine monomers as well as in the HIPE, to optimize the photocuring conditions. The morphology of the obtained monoliths was determined by scanning electron microscopy (SEM) and the hydrophobicity of these materials was determined by water contact angle (WCA). The thermal properties of the monoliths were analyzed by differential scanning calorimetry (DSC) and thermogravimetric analysis (TGA). The compression and hydrocarbon absorption properties were also analyzed.

## 2. Materials and Methods

### 2.1. Materials

**Figure 1:**
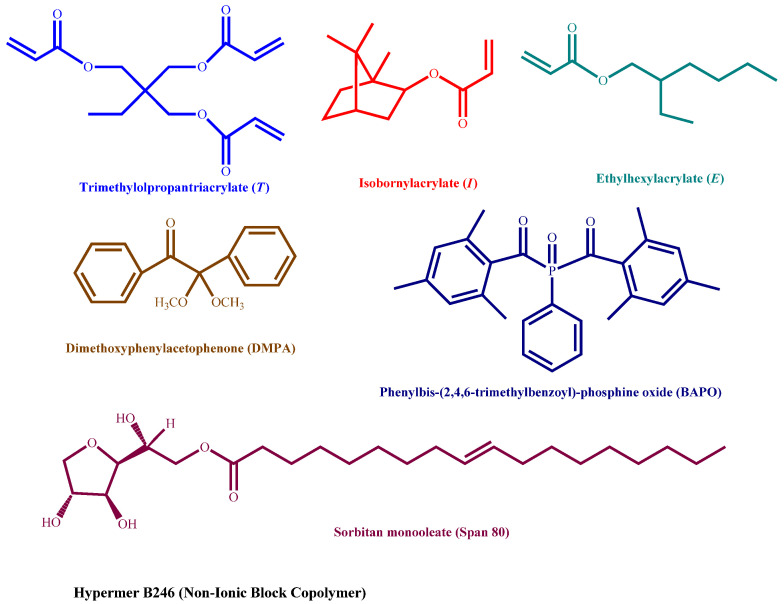
Chemical structures of the compounds used in this study.

Ethylhexyl acrylate (98%) (E), isobornyl acrylate (technical grade) (I), trimethylolpropane triacrylate (technical grade) (T), 2,2-dimethoxy-2-phenylacetophenone (99%) (DMPA), phenylbis-(2,4,6-trimethylbenzoyl)-phosphine oxide (97%) (BAPO), sorbitan monooleate (Span 80, HLB = 4.3) and calcium chloride (97%), were purchased from Merck (Toluca, México) and used as received. Hypermer^TM^ B246-SO-MV (HLB = 6), was kindly donated by the company Croda Mexico (Ciudad de, Mexico). Figure 1 illustrates the chemical structures of noted compounds.

### 2.2. Determination of Kinetics of Photopolymerization of Pristine Acrylate Monomers by Real-Time Fourier Transforms Infrared Spectroscopy (RT-FTIR)

The reactivity of the pristine monomers used to prepare the polyHIPEs was determined using the RT-FTIR technique. This time-resolved methodology measured the decrease in the absorbance of the double bonds of the acrylate groups at 1636 cm^−1^ in a determined time when the photocurable formulation was irradiated with a UV-Vis lamp. The photocurable formulations were prepared by mixing the trifunctional monomer T with the monofunctional monomers I and E, separately and in mixture, in stoichiometric ratios. Table 1 displays the amounts in grams and moles of each component of the formulations. 5 μL of each photocurable mixture was placed between two 3 cm × 3 cm corona-treated PP films. The obtained sandwich was then set in a metal sample holder (held by magnetic strips), that in turn, was vertically positioned within the sample compartment of a Thermofisher Nicolet 6700 FTIR equipment (Thermo Fisher Scientific, Franklin, MA, USA). A Dymax Blue Wave 200 UV-Vis lamp (Dymax, Torrington, CT, USA) was used to perform the experiments. This lamp emits energy in the UVA and visible portion of the spectrum (300–450 nm) (see Figure S1 for emission spectrum of this lamp). The tip of the guide light of the lamp was placed at 45° with respect to the horizontal infrared beam. The distance between the sample and the tip of the UV-Vis lamp was varied between 15 to 20 cm to modulate the intensity of UV light reaching the sample. The data collection rate was 1 spectrum/second. Tests were repeated a minimum of five times for all monomers and the average of these runs is reported. The acrylate conversions were determined by following the band located at 1636 cm^−1^ corresponding to acrylate double bond vibrations, taking the band of the carbonyl group at 1736 cm^−1^ as reference.

### 2.3. Preparation of High Internal Phase Emulsions (HIPEs)

Table 1 shows the amounts of the components of the HIPE formulations. Since the molar ratio between the crosslinker (T) and the monofunctional monomers (E and I) is 1:3 respectively, the nomenclature is stated as T–E–I (1:*x*:*y*) where *x* + *y* = 3, and *x* and *y* represent the molar amounts of monomers E and I. For example, the formulation with T and E is written as T–E (1:3), while the mixed formulation with an equimolar proportion of E and I is written as T–E–I (1:1.5:1.5).

The HIPEs were prepared by mixing the monomers and photoinitiators in an aluminum-foil covered beaker, followed by the addition of the surfactants Hypermer B-246 and Span 80, each at 10% *w*/*w* of the weight of the mixture of monomers. Then, the saline solution was added to the beaker at a dropping rate of 1 mL/min by means of a programmable syringe pump, until it reached 76 ± 1% of the total amount of the HIPE. Over-head mechanical stirring at 500 rpm was continuous during the addition of the saline solution and kept for 10 min after completion. Each formulated HIPE was kept refrigerated (7 ± 1 °C) for 30 min and finally, aliquots of the emulsions were extracted to conduct each one of the different photocuring tests.

### 2.4. Determination of Kinetics of Photopolymerization of HIPEs by Gravimetry

A gravimetry procedure to determine the conversion of acrylate monomers to polyHIPEs at a given time, was performed to compare the theoretical amount of polymer that can be obtained from each formulated HIPE, with respect to the experimentally obtained material. The following procedure was performed to irradiate the prepared HIPEs: 2.5 mL of the HIPE were poured in a glass mold (0.4 cm high, 2.5 cm wide, 2.5 cm long) to irradiate the prepared HIPEs. The mold was horizontally positioned on a laboratory jack, allowing control of the intensity from the UV lamp to the sample, by varying the distance between the sample and the lamp (10–20 cm). The UV light intensity reaching the sample was measured using a UVEXS PM 600 control cure radiometer (Uvexs Inc. North Bend, OR, USA). The emulsion contained within the mold was exposed to constant radiation at different exposure times. A set of samples of the same emulsion were irradiated with the UV-Vis lamp, separately and at various times. After the specified time, the lamp was turned off and the samples were removed from the mold using a steel blade and washed at once with acetone and then extracted with ethanol for 24 h, in a Soxhlet equipment, to remove any unreacted monomers and surfactants. Finally, the material was dried under vacuum conditions until reaching a constant weight. This procedure was performed for all formulated HIPEs, at different radiation intensities (from 30 to 180 mW/cm^2^) to develop an experimental matrix (radiation intensity-exposure time) that allows finding the optimal photocuring conditions for the porous monoliths preparation.

### 2.5. Scanning Electron Microscopy (SEM)

The morphological analysis of the obtained monoliths was performed using a JEOL JCM 6000 scanning electron microscope (JEOL, Peabody, MA, USA). Samples were Au-Pd coated by chemical vapor deposition and then the analysis was performed under high vacuum conditions at 15 kV. All micrographs taken correspond to 4000× of magnification.

### 2.6. Analysis of Surface Area and Pore Size Distribution

The surface area analysis was determined using the BET method [52]. The measurements were conducted in a Quantachrome Nova 4200e surface area analyzer (Quantachrome Instruments, Ashland, VA, USA) at 77.35 °K. The samples were treated prior to the analysis at room temperature for 12 h with high vacuum for degassing and surface cleaning. Surface area measurements used 11 points collected over 0.05 to 0.3 P/P_0_. The samples were analyzed by duplicated and the average is reported. The surface porosity of the monoliths was determined using the BJH method.

### 2.7. Water Contact Angle (WCA)

The WCA was determined using a Ramé-Hart Inc. 100-00 goniometer (Succasunna, NJ, USA) fitted with a Schott-Fostec, LLC lamp (Mainz, Germany). The analyses were performed on both, the surface of the photocured thin film of pristine acrylates placed on a microscope slide, and on the surface of the obtained monoliths. For each surface analysis, 10 μL of deionized water drops were placed at different points on each sample (both for the films on the slides and for the monoliths). Then the images were focused on a screen and analyzed at once to know the value of WCA. Reported values are the averages of different measurements for each surface.

### 2.8. Compression Properties

The MTS Criterion ^TM^ Electromechanical Universal Test System Model 43 using a 50 Kg load cell with a compression speed of 1 mm/min was used to perform the analysis. Test specimens of 1 cm^2^ with a width of 0.4 cm were used.

### 2.9. Thermogravimetric Analysis (TGA)

The TGA analysis was performed using a TA Instruments Q500 thermal analyzer (New Castle, DE, USA) from 30 to 600 °C at a heating rate of 10 °C min^−1^ in a high-purity nitrogen atmosphere.

### 2.10. Differential Scanning Calorimetry (DSC)

The obtained monoliths were analyzed in a TA Instruments Q 200 differential scanning calorimeter (New Castle, DE, USA) to characterize the glass transition temperature (T_g_). Samples of 8–10 mg were accurately weighed, then, the equipment was set to heat the samples from –20 to 150 °C at a heating rate of 10 °C min^−1^ in a nitrogen atmosphere. The T_g_ was found from the second heating curve.

### 2.11. Determination of Absorption Properties of Hydrocarbons

The prepared polyHIPEs were evaluated to determine their absorbing properties of hydrocarbons such as hexane, diesel, toluene and chloroform. The polyHIPEs (0.5 g) were added separately to beakers containing 10 mL of the solvent and left for 10 min, allowing the swelling of the material. After this period, the samples were withdrawn of the solvent and the excess of the solvent was wiped and the swollen polyHIPE was accurately weighed to determine the % absorption. Thereafter, the materials were dried for 24 h to constant weight and the cycle absorption-drying-absorption was repeated 10 times.

## 3. Results and Discussion

### 3.1. Determination of Kinetics of Photopolymerization of Pristine Monomers

**Figure 2:**
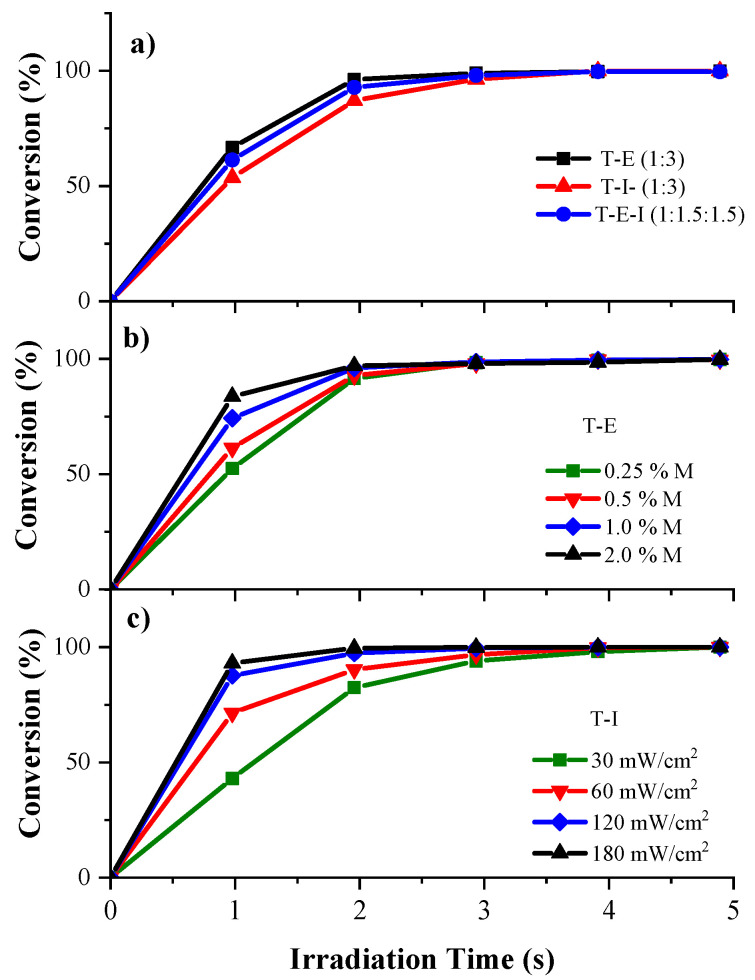
Kinetics of photopolymerization of pristine acrylates using the RT-FTIR technique: (**a**) comparison of the reactivity of the formulations T–E (1:3), T–I (1:3) and T–E–I (1:1.5:1.5), at 0.5 mol% of DMPA and 0.5 mol% of BAPO at 30 mW/cm^2^ of UV-Vis light intensity; (**b**) effect of the concentration of the mixture of photoinitiators DMPA:BAPO on T–E formulation at 60 mW/cm^2^; (**c**) effect of the UV-Vis light intensity on the photopolymerization rate of the formulation T–I at 0.5% mol of photoinitiators.

As a first step to prepare the polyHIPEs, we assessed the reactivity of the selected monomers. Though styrene and divinylbenzene were evaluated, they were not reactive enough to photopolymerize in a reasonable frame of time, due to the highly stabilized propagating radicals produced. Therefore, it was decided to use acrylates whose high reactivity is well known. A trifunctional monomer T was used as a cross-linker alongside monofunctional monomers I and E. The monomer I presents low viscosity and low shrinkage during polymerization and impart a rigid structure to the monolith while polyHIPEs derived from E are soft materials. The reactivity of the pristine acrylate monomers was found by using the RT-FTIR technique, to determine if the photocurable system allows a rapid formation of interconnected porous in the monoliths. The RT-FTIR technique is a reliable technique sensitive enough to analyze the kinetics of ultrafast photopolymerizations [53]. The progress of the photopolymerizations was determined by measuring the decrease in the absorbance in the characteristic band of double bonds at 1636 cm^−1^. Figure 2a shows the conversion versus irradiation time curves of the formulations T–I and T–E, both in a 1:3 ratio and of the mixture T–E–I in a 1: 1.5:1.5 ratio.

All three formulations displayed a very rapid photopolymerization, achieving conversions of 90% in 2 s, when irradiated with UV light intensity of 30 mW/cm^2^ and a concentration of 0.5 mol% of both, BAPO and DMPA. A quantitative conversion was accomplished after 3 s in all cases. The formulation T–E (1:3) displayed a higher photopolymerization rate than the formulation T–I (1:3) while the formulation T–E–I (1:1.5:1.5) displayed an intermediate rate. The slightly lower reactivity of the formulation T–I was attributed to the steric hindrance of the bicyclic ring of I. The effect of the concentration of the photoinitiators was evaluated in the formulation T–E (Figure 2b). There was a significant increment in the conversion when the concentration of the photoinitiators augmented from 0.25 to 2.0 mol%, passing from 52% to 84% conversion during the first second of irradiation, at a UV light intensity of 30 mW/cm^2^. The increase in reactivity is due to the higher concentration of initiating species.

Another parameter that was studied to increase the reactivity of the photocurable formulations was the UV-Vis light intensity. The formulation T–I was selected to perform this study (Figure 2c). By increasing the intensity of the UV light and keeping constant the concentration of the photoinitiators (0.5 mol%), the conversion of the acrylate groups of the formulation T–I was augmented from 43% to 93% in the first second of irradiation when passing from 30 to 180 mW/cm^2^ and quantitative conversion in both cases after 4 s. This behavior can be attributed to the termination reactions such as coupling of macroradicals to render neutral species of higher molecular weights, and to the intermolecular hydrogen abstraction to produce inactive double bond-terminated chains. These reactions occur more rapidly when fewer reactive species are produced at low light intensities. Doubling the light intensity starting from 30 mW/cm^2^, resulted in a significant increase in the photopolymerization rate. However, after 120 mW/cm^2^ no further augment was detected. These results confirm the high reactivity of the monomers selected to prepare the polyHIPEs. It can be concluded from this part that augmenting the UV light intensity is a more cost-effective means to increase the reactivity of our photocurable system considering the cost of photoinitiators.

### 3.2. Preparation of the Photocurable PolyHIPEs

PolyHIPEs are generally obtained by thermal radical polymerization of styrenic or acrylate monomers, mainly. These thermal processes use temperatures in the range of 50 to 70 °C and reaction times that go from 8 to 24 h, depending on the types of monomers. In this study, we aimed to develop a faster and efficient method to polymerize HIPEs by using a photopolymerization technique. A mixture of two surfactants, Span 80 (HLB = 4.3) and Hypermer B246 (HLB = 6.0), was used due to the instability of the formulations when only one of the surfactants was used. Different tests varying the ratio of both surfactants revealed that a 10% *w*/*w* of both surfactants yielded the best stability results.

**Figure 3:**
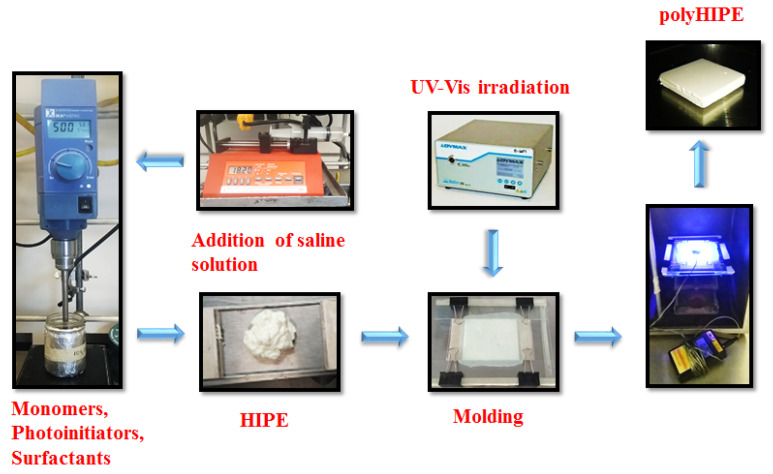
Methodology to obtain the photocurable polyHIPEs.

The procedure to obtain the photocurable polyHIPEs is schematized in Figure 3. The obtained HIPEs were mayonnaise-like liquids. All HIPEs remained stable during the handling process. Then, the HIPE was poured into a glass mold and irradiated with a UV-Vis lamp. To photocure the w/o emulsion, a 1:1 mixture of photoinitiators was used: a UV light absorber such as DMPA and a blue light absorber such as BAPO—405 nm—with the aim to enhance the radiation absorption. Both photoinitiators are of the Norrish I type. After photolysis of both photoinitiators, benzoyl radicals are produced, which react with the double bond of the acrylates initiating the radical photopolymerization (see Figure S2 for photoinitiation mechanisms). The photocurable formulations were irradiated for 20 min to obtain the polyHIPEs. The formulations were irradiated at the specified times and then the obtained monoliths were purified by Soxhlet extraction using ethanol to remove surfactants and unreacted monomers.

### 3.3. Kinetics of Photopolymerization of the HIPEs

**Figure 4:**
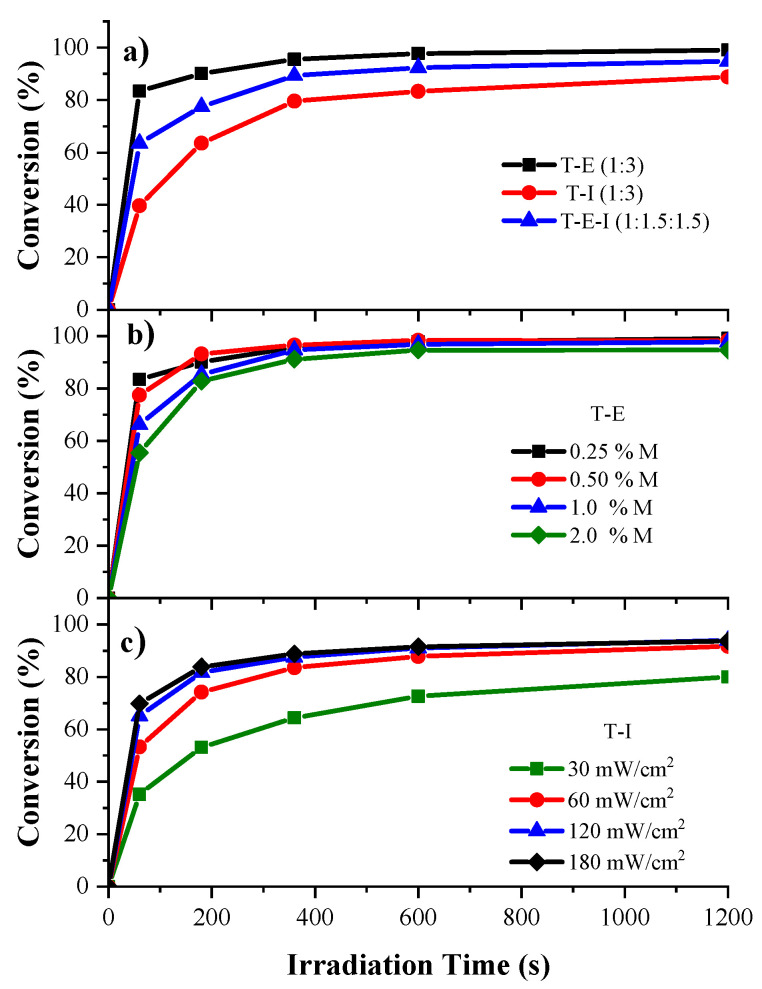
Kinetics of photopolymerization of the acrylate HIPEs using the gravimetry technique: (**a**) comparison of the reactivity of the formulations T–E (1:3), T–I (1:3) and T–E–I (1:1.5:1.5) at 0.5 mol% of DMPA and 0.5 mol% of BAPO at 30 mW/cm^2^ of UV-Vis light intensity; (**b**) effect of the concentration of the mixture of photoinitiators DMPA:BAPO on T–E formulation at 60 mW/cm^2^; (**c**) effect of the UV-Vis light intensity on the photopolymerization rate of the formulation T–I at 0.5% mol of photoinitiators.

The kinetics of photopolymerization of the HIPEs when irradiated with UV light, were determined by means of gravimetry (Figure 4). Samples of the same formulation were irradiated at various times and the amount of polyHIPEs produced was found.

The photocurable formulations of HIPEs displayed the same pattern of reactivity found for the pristine acrylates, though with longer times to achieve the same conversions. The formulation T–E (1:3) was the more reactive, followed by the mixture T-E-I (1:1.5:1.5) and lastly by the formulation T–I (1.3), though the differences in reactivity were more marked (Figure 4a) compared with the pristine monomers. Conversions of 83%, 63% and 40% of the acrylate groups were achieved, respectively, after 60 s of irradiation, followed for a deceleration of photopolymerization rate, due to a diffusion effect of the free radicals in the highly viscous media of the HIPE and to the scattering of the UV light due to opacity of the emulsion. Final conversions of 99%, 94% and 88% were achieved after 1200 s.

All formulations displayed a similar photopolymerization rate, when the concentration of photoinitiators was varied in the T–E (1:3) HIPEs (Figure 4b), though the HIPEs with 2.0 mol% of both photoinitiators displayed lower reactivity, while the formulation with 0.25 mol% of both photoinitiators showed the highest. Due to the high reactivity of the T-E formulations, conversions of 99%, 98%, 98% and 94% were attained for the formulations with increasing concentration of photoinitiators, after 1200 s. This can be attributed to the rapid build-up of the crosslinked network in the highly viscous media of the HIPE, due to the high concentration of free radicals, hindering the propagation reactions, which results in a decrease in the photopolymerization rate [54,55]. A disadvantage of using high concentration of photoinitiators is the increase in the levels of yellowing. Figure 4c depicts the comparison of the kinetic curves of the formulation of the T–I (1:3) HIPEs when the UV-Vis light was varied from 30 to 180 mW/cm^2^. A similar behavior to the one observed for the pristine monomers occurred, with a relatively low reactivity at 30 mW/cm^2^ and progressively increasing the photopolymerization rate by augmenting the light intensity up to 120 mW/cm^2^ from which further increase did not result in higher reactivity. Final conversions of 80% and 91% for the samples irradiated with 30 and 60 mW/cm^2^ were obtained, while for the samples irradiated with 120 and 180 mW/cm^2^ the conversion was of 93% in both cases, after the end of the irradiation time. The increase in the reactivity could be due to the increase in the light penetration depth and to a slight augment of the temperature due to the high intensity of the UV light, which could reduce the viscosity of the HIPE, enhancing the mobility of the free radicals. Thus, increasing the light intensity is a more effective means to augment the reactivity of the photocurable HIPEs with optimum intensity of 60 mW/cm^2^.

### 3.4. Thermal Analysis of Monoliths by DSC and TGA

**Figure 5:**
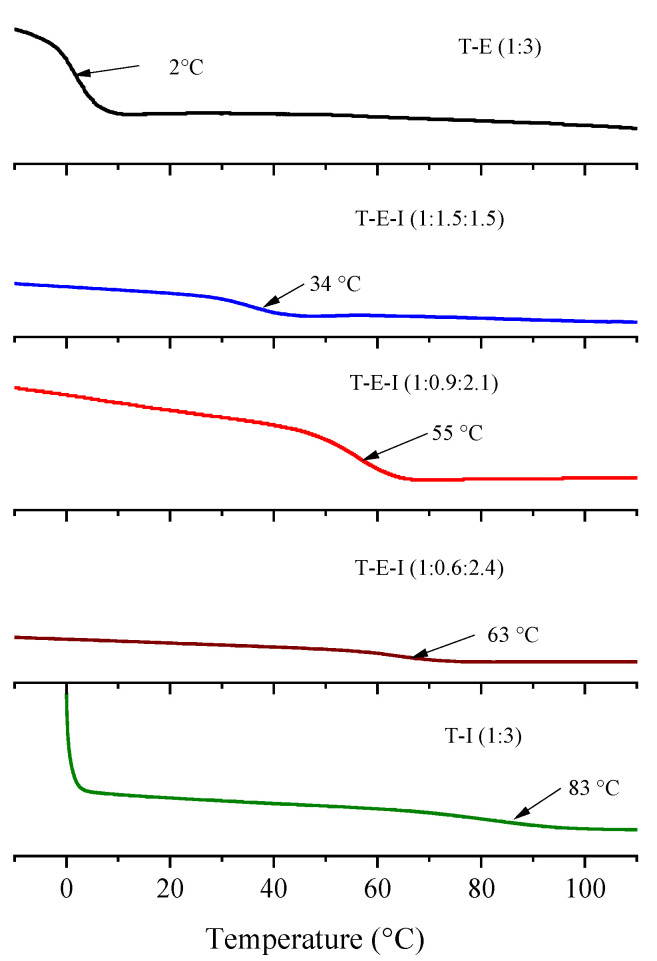
Comparison of the T_g_ of the obtained polyHIPEs.

The obtained monoliths were analyzed by DSC to determine the glass transition temperature (T_g_) of the obtained polyHIPEs. Figure 5 depicts the thermograms of the samples. The monolith derived from the formulation T–I was a hard and brittle material with a T_g_ of 85 °C while the T–E polyHIPE was soft with a T_g_ of 2 °C. The relatively high T_g_ of the T–I monolith is due to the bulky side group of I. It was reasoned that by combining both monomers E and I with T, the properties of toughness and T_g_ of the monoliths could be modulated, therefore, formulations with different ratios of E and I, keeping higher the concentration of the latter, to increase the T_g_, were prepared: T–E–I (1: 1.5:1.5); T–E–I (1: 0.9:2.1); T–E–I (1:0.6:2.4). The formulation with 50% of each monofunctional monomer displayed a T_g_ of 32 °C, and by increasing the ratio of I, the T_g_’s augmented to 55 and 63 °C, obtaining stronger and tougher materials in the last two cases. No other transitions were observed in the range of temperatures studied (–10 to 120 °C).

**Figure 6:**
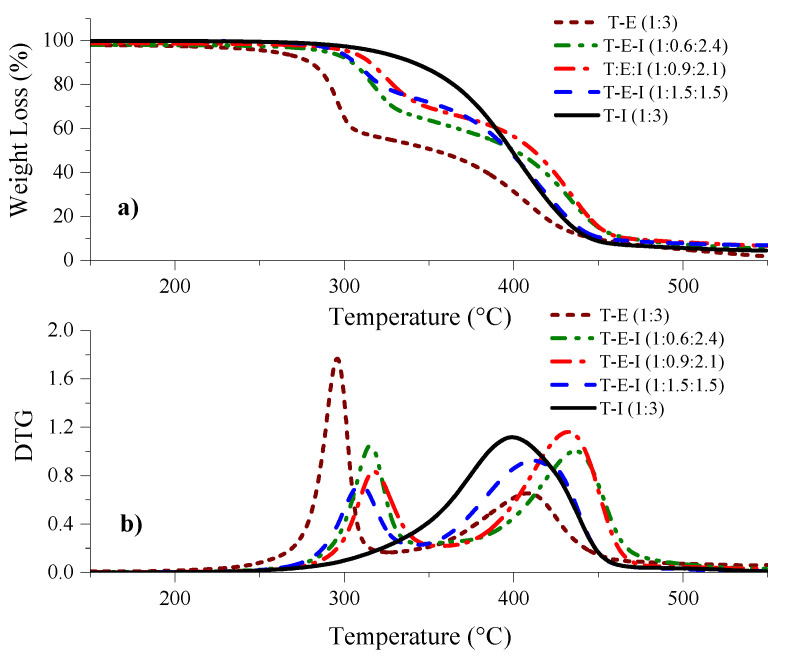
Results of TGA analysis of polyHIPEs: (**a**) weight loss vs. temperature, (**b**) derivative curves versus temperature.

The thermal stability of the polyHIPEs was determined using the TGA technique. Results are shown in Figure 6. Observations show that the T–E derived monolith displayed an onset of degradation—the temperature at which 5% of the mass of the polymer was lost—of 257 °C while for that of the T–I was 319 °C. Increasing the concentration of I in the monoliths derived from T–E–I resulted in intermediate onset temperatures between 289 and 302 °C (see Table 2). The DTG curves showed that the degradation of the T–I monoliths proceeded in one stage, while the degradation displayed two stages in the cases of the monoliths that included E. It is well known that degradation of polyacrylates proceeds via a β-elimination process that results in the scission of the acrylate network [56].

### 3.5. Morphology of the PolyHIPEs by SEM

**Figure 7:**
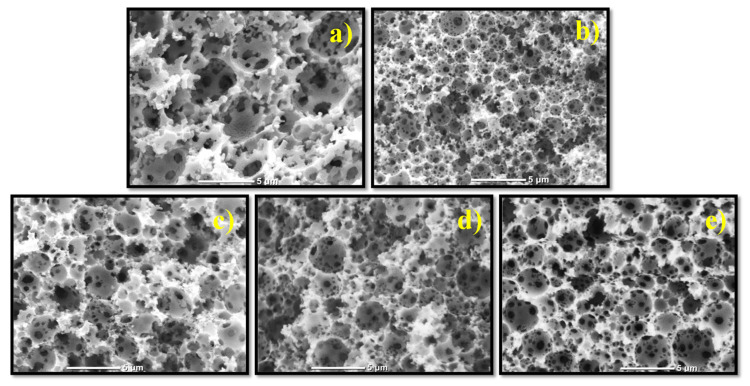
Morphology of obtained monoliths (**a**) T–E (1:3); (**b**) T–I, (1:3); (**c**) T–E–I (1: 1.5:1.5); (**d**) T–E–I (1: 0.9:2.1); (**e**) T–E–I (1: 0.6:2.4).

SEM micrographs of all obtained monoliths displayed a well-defined characteristic morphology with large spherical voids with interconnecting windows. The scale bar in all cases is 5 μm. Figure 7 illustrates that the monolith derived from the T–I (1:3) HIPE displayed lower void diameter and thinner walls than T–E (1:3), while the monoliths derived from the formulations T–E–I showed an intermediate size.

The variation in the pore size of the different monoliths relies on the slightly lower stability of the emulsion of T–E (1:3) during the photopolymerization, compared against that of T–I (1:3). This results in coalescence of the internal phase, increasing the size of the pore. Prior research reports that droplet size decreases with increasing emulsion stability since the surface energy per unit area is lower [57]. By combining I, E and T at different ratios, intermediate values of void diameters were observed. These results show that a high polymerization rate is not enough to maintain the template of the emulsion during the photopolymerization, and stability of the emulsion plays an important role to control the size of the pores in the monoliths.

The monoliths were also analyzed by the BET technique to find the surface area and pore size distribution. The corresponding data are displayed in Table 3. The monolith derived from the formulation T–I displayed the highest surface area (16 m^2^/g) which is consistent with the SEM micrographs (Figure 7b), where it displayed the smaller size of pores, resulting in higher surface area. This is also reflected in a higher total volume of pores (0.059 cm^3^/g). The T–E monolith showed a surface area of 9.4 m^2^/g and a total volume of pores of 0.040 cm^3^/g. The formulations T–E–I with different ratios, displayed intermediate surface areas while the total volume of pores did not follow a consistent pattern.

### 3.6. Water Contact Angle (WCA)

**Figure 8:**
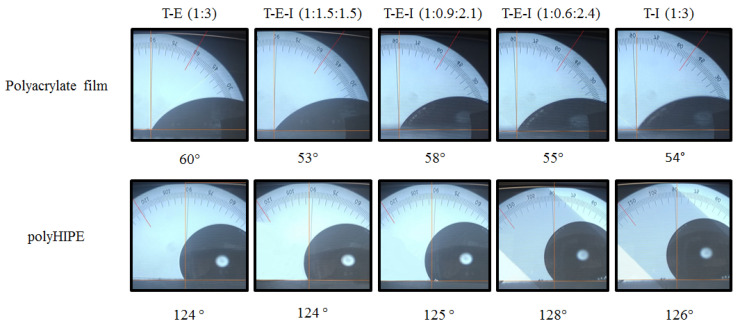
Comparison of Water Contact Angle (WCA) of photopolymerized films of polyacrylates derived from T–E, T–I and T–E–I and their corresponding polyHIPES.

An interesting phenomenon was found for the studied polyHIPEs. The polyacrylates are polar species due to the ester group, which was corroborated by measuring the WCA of a thin film of photopolymerized pristine acrylates (53°–60°). However, the monoliths obtained by photopolymerizing the corresponding HIPE, displayed WCAs of 124–128° as shown in Figure 8.

These high WCAs imply that the obtained polyHIPEs could be classified as hydrophobic materials according to the classification proposed by the Sweden Transmission Research Institute (STRI) [58] even if the chemistry of the material points out that they are polar. This complex behavior was attributed to water repellency due to the roughness of the surface of the obtained porous monolith. The high surface area of the monoliths increases the surface energy, resulting in a more hydrophobic surface [59,60,61]. Moreover, the pores in the surface of the polyHIPE can form air pockets under the water droplet that prevents a complete settling on the surface of the porous material, leading to high values of WCA. Another factor involved is the high surface tension of the water (72 mN/m), which resists the penetration of water in the pores of the monolith. This phenomenon is somehow related to the well-known “Lotus Effect” [62] where the roughness of lotus leaves impedes their wetting (see Video S1 for video of hydrophobic properties of the obtained polyHIPEs).

### 3.7. Mechanical Properties of Compression

**Figure 9:**
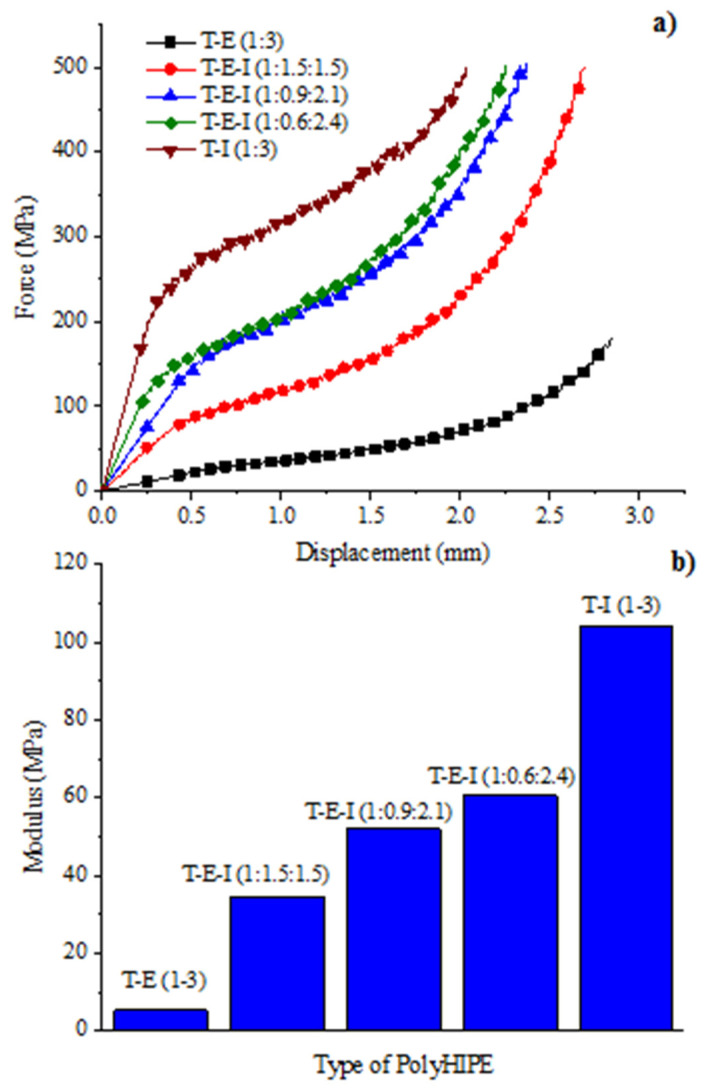
Compression properties of the obtained polyHIPEs: (**a**) The comparison of displacement vs. force curves of the different polyHIPEs, (**b**) the comparison of the moduli of polyHIPEs.

The results of the compression analysis of the obtained monoliths are depicted in Figure 9a showing the substantial differences between the porous materials derived from T–I (1:3) and T–E (1:3). In the first case, the compression curve displayed three zones (domains) when subjected to a pressure force. When moving from one area to another, the domains are denoted by changes in the slope of the curve. The first domain is related to the elastic behavior of the material where the compressibility modulus is found. This domain was characterized for a displacement up to 0.4 mm when pressure exceeds 240 MPa. The change in slope is equivalent to a deformation of 5–7% indicating that the monolith is very rigid. The second domain begins when the compression exceeds the elastic limit of the material resulting in deformation or fracture, in an irreversible way, starting from the weakest points of the structure to the most robust. As compression continues, the curve displays a stable behavior, despite being oscillatory due to the accumulation-release energy processes as a consequence of compression and fracturing of the monolith. This stable behavior is maintained until the polymer is compressed up to 1.75 mm at 400 MPa of compression force. The third domain corresponds to the compaction of the polymer. The change in slope could be attributed to the accumulation of fragments that are separated from the main structure and are enclosed within the remaining scaffold, which begins to apply added resistance to that of the monolith itself.

In the case of the polyHIPE derived from T–E (1:3), only one change in the slope of the curve, due to the softness of the material, was observed. This sample was compressed up to 2.8 mm (equivalent to a 70% deformation) applying a force of only 170 MPa. The shape of the compression curve for this sample allows the differentiation between the compression and compaction zones (~1.8–1.9 mm); however, it is not possible to distinguish the slope between the elastic and the inelastic domains. The variations in the applied force are not oscillatory as in the case of the T–I polyHIPE, therefore the T–E polyHIPE is composed of a reversible elastic domain even at 1.6 mm of compression (40% deformation) indicating that the materials is the most flexible (softest) of the series of the synthesized monoliths.

The T–E–I polyHIPEs displayed an intermediate behavior, where the three zones are clearly marked as in the case of T–I. However, by increasing the concentration of E in the formulation, the first domain was progressively reduced, while the third domain augmented significatively, indicating an increase in softness of the monolith, which agrees with the study of the T_g_ determined by DSC.

The compression moduli (*K*) of the obtained polyHIPEs were obtained from the first domain of the compression curves and are shown in Figure 9b. The compression modulus expresses the resistance of a material to its uniform elastic compression and allows knowing the pressures required to cause a decrease in a given volume of matter. The results show that *K* for the T–E (1:3) and T–I (1:3) polyHIPEs were of 5.26 MPa and 103 MPa, respectively, which shows that the former is almost 20 times softer than the latter. In the mixed formulations T–E–I it was found that by increasing the proportion of *I* in the polyHIPEs, *K* augmented reaching a value of 60.6 MPa for the formulation T-E-I (1:0.4.2.6). This indicates that the toughness of the porous material can be modulated with the right proportion of E, to obtain materials with excellent mechanical properties and the adequate Tg.

### 3.8. Hydrocarbon Absorption Properties and Study of the Reusability of the PolyHIPEs

Table 4 shows the average absorption of the 10 cycles for each prepared polyHIPE. All the prepared porous materials displayed excellent properties of absorption of hydrocarbons especially of chloroform, which was absorbed in a higher percentage than the other hydrocarbons. This can be attributed to its polar nature that makes it more compatible with the polar polyacrylates. The theoretical percentage of absorption is 300%, considering that the HIPE was composed of 75% in volume, of the internal phase, which was used as a template. In most of the cases, this value was achieved and surpassed.

The T–I (1:3) and T–E–I (1:0.6.2.4) polyHIPEs displayed elevated levels of absorption of chloroform, of 1570% and 1325%, respectively. However, this was due to the fragmentation of those samples when absorbing the solvent, due to their high brittleness, which resulted in higher surface area with the consequent augment in its absorption properties. The polyHIPEs derived from T–E, T–E–I (1:1.5:1.5) and T–E–I, (1:0.9:2.1) displayed absorptions of 823%, 806% and 778% respectively, though, in all these cases the polyHIPEs were not fragmented even at this high absorption of chloroform, which confirms the affinity of the polar solvent with the polymeric matrix, as well as the toughness of the T–E–I (1:1.5:1.5) and of T–E–I (1:0.9:2.1) polyHIPEs, though the latter has a T_g_ of 55 °C against 33 °C of the former.

Toluene was the second hydrocarbon that was more highly absorbed. The same order of capability of absorption as in the case of chloroform, was observed, with T–I (1:3) and T–E–I (1:0.4:2.6) as the best absorbents with 595% and 550% absorption, respectively. Nonetheless, both materials also underwent fragmentation during the swelling. The T–E (1:3), T–E–I (1:1.5:1.5) and T–E–I (1:0.9:2.1) polyHIPEs absorbed 448%, 440% and 378% of toluene. Hexane showed slightly lower absorption than toluene. However, in this case, it was observed that the polyHIPEs that contained E displayed better absorption than T–I (250%). For example, the T–E (1:3), T–E–I (1:1.5:1.5), T–E–I (1:0.9:2.1) and T–E–I (1:0.6:2.4) polyHIPEs showed absorptions close or above 300%, which agree with the theoretical internal volume. This could be related to the higher affinity of hexane with the aliphatic hydrocarbonated chain of E than with the bicyclic ring of I.

Diesel displayed the lowest absorption of the studied polyHIPEs, ranging from 158% for T–I (1:3) to 265% for T–E (1:3), and intermediate values for the mixed polyacrylates. This was attributed, as in the case of hexane, to the non-polar character of the hydrocarbon as well as to the higher density of diesel (0.85 kg/L) [63] which is 15–20% higher than that of hexane, difficulting the capillary absorption of this hydrocarbon through the porous structure of the polyHIPE. A related study of polyacrylate-derived photo-polyHIPEs [64] reported absorptions of 275 g/g of toluene, 380 g/g of chloroform, diesel 150 g/g and gasoline 140 g/g.

Based on these results it can be summarized that all prepared polyHIPEs displayed excellent properties of absorption of hydrocarbons especially of chloroform and toluene, though in the case of hexane and diesel the polyHIPEs that displayed moderate absorption properties, they were able to absorb 300% and 200% their weight, respectively. Therefore, the T–E–I, (1:0.9:2.1) polyHIPE can be selected as the best option to be used as hydrocarbon organic solvent absorber, considering both its physical and mechanical properties as well as its capability of absorption.

## 4. Conclusions

A cost-effective process to prepare porous polyacrylate materials with properties of organic solvents absorption, was developed. The polyHIPEs were obtained via a photopolymerization technique, irradiating formulations of the acrylic monomers T, E and I, for 20 min at 60 mW/cm^2^ using 1 mol% of both photoinitiators BAPO and DMPA. The obtained polyHIPEs displayed the classical morphology with voids whose diameters were in the range of 2–5 μm, with interconnecting windows. By combining the acrylic monomers, it was possible to modulate the toughness of the porous polyacrylates. The prepared polyHIPEs displayed excellent properties of absorption of hexane, toluene, diesel and especially of chloroform, finding that the best absorbing polyHIPE was that derived from the formulation T–E–I (1:0.9:2.1), which displayed the best properties of toughness and absorption.

## Figures and Tables

**Table 1:** Composition of the formulations for each studied photocurable HIPE.

Phase/Samples			Monomers			Photoinitiators *	
	Nomenclature (Molar Ratios)	E:I %ratio	T (g) (mmol)	E (g) (mmol)	I (g) (mmol)	DMPA (g) (mmol)	BAPO (g) (mmol)
Organic phase	T–E (1:3)	100:0	2.092	2.908	0	0.152	0.257
			4.888	14.665	0	0.592	0.605
	T–E–I (1:1.5:1.5)	50:50	2.062	1.433	1.505	0.150	0.254
			4.817	7.226	7.226	0.584	0.596
	T–E–I (1:0.9:2.1)	30:70	2.050	0.855	2.095	0.149	0.252
			4.789	4.311	10.059	0.581	0.593
	T–E–I (1:0.6:2.4)	20:80	2.044	0.568	2.388	0.148	0.251
			4.776	2.866	11.463	0.579	0.591
	T–I (1:3)	0:100	2.032	0	2.968	0.148	0.250
			4.749	0	14.247	0.576	0.587
Aqueous phase			18.4 mL (3% *w*/*w* CaCl_2_ solution)				
Surfactants			Hypermer B246 = 0.5 g/Span 80 = 0.5 g				

* These values correspond to 1% molar with respect to the total amount of double bonds.

**Table 2:** Thermogravimetric data of the thermal degradation of obtained polyHIPEs.

Sample	T _5%_ ^a^	T_max_ ^b^
T–E (1:3)	257	296, 411
T–E–I (1:1.5:1.5)	289	310, 414
T–E–I (1:0.9:2.1)	296	317, 434
T–E–I (1:0.6:2.4)	302	314, 436
T–I (1:3)	319	398

^a^ Temperature of 5 % weight loss, ^b^ Temperature of maximum degradation rate.

**Table 3:** Results of the BET analysis of the polyHIPEs.

Sample	BET Area (m^2^/g)	Total Volume of Pores (cm^3^/g)
T–E (1:3)	9.4	0.040
T–I (1:3)	16	0.059
T–E–I (1:1.5:1.5)	11.2	0.029
T–E–I (1:0.9:2.1)	11.5	0.028
T–E–I (1:0.6:2.4)	12.0	0.037

**Table 4:** Absorption properties of organic solvents of the prepared polyHIPEs.

Sample	Hexane *	Diesel *	Toluene *	Chloroform *
T–E (1:3)	332	265	448	823
T–I (1:3)	250	158	595	1570
T–E–I (1:1.5:1.5)	321	253	440	806
T–E–I (1:0.9:2.1)	306	236	378	778
T–E–I (1:0.6:2.4)	294	206	550	1325

* Average of % absorption of organic solvents during 10 cycles absorption-drying-absorption.

## Supplementary Materials

The following are available online at https://www.mdpi.com/article/10.3390/polym13203497/s1, Figure S1: Emission spectrum of Blue wave dymax UV-Vis lamp, Figure S2: (a) Mechanism of photoinitiation of dimethoxyphenyl acetophenone (DMPA), (b) Mechanism of generation of radical and initiation of photopolymerization of acrylates using BAPO. Video S1: Video of hydrophobic properties of polyHIPE derived from formulation T:E:I (1:0.9:2.1).

## References

[1] Qiu S., Ben T. (2015). Porous Polymers: Design, Synthesis & Applications.

[2] Kircher L., Theato P., Cameron N.R., Theato P., Klok H.A. (2013). Functionalyzation of porous polymers from high internal phse emulsions and their applications. Functional Polymers by Post-Polymerization Modification.

[3] Wu D., Xu F., Sun B., Fu R., He H., Matyjaszewski K. (2012). Design and Preparation of Porous Polymers. Chem. Rev..

[4] Ahmed D.S., El-Hiti G.A., Yousif E., Ali A.A., Hameed A. (2018). Design and synthesis of porous polymeric materials and their applications in gas capture and storage: A review. J. Polym. Res..

[5] Wu J., Xu F., Li S., Ma P., Zhang X., Liu Q., Fu R., Wu D. (2019). Porous Polymers as Multifunctional Material Platforms toward Task-Specific Applications. Adv. Mater..

[6] Silverstein M.S. (2014). PolyHIPEs: Recent advances in emulsion-templated porous polymers. Prog. Polym. Sci..

[7] Zhang T., Sanguramath R.A., Israel S., Silverstein M.S. (2019). Emulsion Templating: Porous Polymers and beyond. Macromolecules.

[8] Cameron N.R., Sherrington D.C. (2006). High Internal Phase Emulsions (HIPEs)—Structure, Properties and Use in Polymer Preparation. Adv. Polym. Sci..

[9] Princen H.M. (1983). Rheology of Foams and Highly Concentrated Emulsions, I. Elastic Properties and Yield Stress of a Cylindrical Model System. J. Colloid Interface Sci..

[10] Pulko I., Krajnc P. (2017). Porous Polymer Monoliths by Emulsion Templating. Encyclopedia of Polymer Science and Technology.

[11] Aronson M.P., Petko M.F. (1986). High Internal Phase Emulsions. U.S. Patent.

[12] Park C.I., Cho W.-G., Lee S.J. (2003). Emulsion stability of cosmetic creams based on water-in-oil high internal phase emulsions Emulsion stability of cosmetic creams based on water-in-oil high internal phase emulsions. Korea Australia Rheol. J..

[13] Cameron N.R. (2003). Polymerized High Internal Phase Emulsion Monoliths. Monolithic Materials: Preparation, Properties and Applications.

[14] Butler R., Hopkinson I., Cooper A.I. (2003). Synthesis of Porous Emulsion-Templated Polymers Using High Internal Phase CO2-in-Water Emulsions. J. Am. Chem. Soc..

[15] Livshin S., Silverstein M.S. (2008). Crystallinity and Cross-Linking in Porous Polymers Synthesized from Long Side Chain Monomers through Emulsion Templating. Macromolecules.

[16] Tan L., Wang K., Yang Y., Li Q., Lin Z., Yang Z., Zhang A. (2017). Organic Porous Polymer Materials: Design, Preparation, and Applications. Polymer-Engineered Nanostructures for Advanced Energy Applications.

[17] Desforges A., Arpontet M., Deleuze H., Mondain-Monval O. (2002). Synthesis and functionalisation of polyHIPE^®^ beads. React. Funct. Polym..

[18] Gokmen M.T., Van Camp W., Colver P.J., Bon S.A., Du Prez F.E. (2009). Fabrication of porous ‘clickable’ polymer beads and rods through generation of high internal phase emulsion (HIPE) droplets in a simple microfluidic device. Macromolecules.

[19] Whitely M.E., Robinson J.L., Stuebben M.C., Pearce H.A., McEnery M.A., Cosgriff-Hernandez E. (2017). Prevention of Oxygen Inhibition of PolyHIPE Radical Polymerization Using a Thiol-Based Cross-Linker. ACS Biomater. Sci. Eng..

[20] Krajnc P., Leber N., Štefanec D., Kontrec S., Podgornik A. (2005). Preparation and characterisation of poly(high internal phase emulsion) methacrylate monoliths and their application as separation media. J. Chromatogr. A.

[21] Thunhorst K.L., Gehlsen M.D., Wright R.E., Nelson E.W., Koecher S.D., Gold D. (2003). Foams Made By Photopolymerization of Emulsions. U.S. Patent.

[22] Barby D., Haq Z. (1985). Low Density Porous Cross-Linked Polymeric Materials and Their Preparation and Uses as Carriers for Included Liquids. U.S. Patent.

[23] Pierre S.J., Thies J.C., Dureault A., Cameron N.R., van Hest J.C.R., Carette N., Michon T., Weberskirch R. (2006). Covalent enzyme immobilization onto photopolymerized highly porous monoliths. Adv. Mater..

[24] Cummins D., Wyman P., Duxbury C.J., Thies J. (2007). Synthesis of Functional Photopolymerized Macroporous PolyHIPEs by Atom Transfer Radical Polymerization Surface Grafting. Chem. Mater..

[25] Cummins D., Duxbury C.J., Quaedflieg P.J.L.M., Magusin P.C.M.M., Koning C.E., Heise A. (2009). Click chemistry as a means to functionalize macroporous PolyHIPE. Soft Matter..

[26] Gong X., Wen W., Sheng P. (2009). Microfluidic Fabrication of Porous Polymer Microspheres: Dual Reactions in Single Droplets. Langmuir.

[27] Lovelady E., Kimmins S.D., Wu J., Cameron N.R. (2011). Preparation of emulsion-templated porous polymers using thiol-ene and thiol-yne chemistry. Polym. Chem..

[28] Caldwell S., Johnson D.W., Didsbury M., Murray B.A. (2012). Degradable emulsion-templated scaffolds for tissue engineering from thiol-ene photopolymerisation. Soft Matter..

[29] Kimmins S.D., Wyman P., Cameron N.R. (2012). Photopolymerised methacrylate-based emulsion-templated porous polymers. React. Funct. Polym..

[30] Schüler F., Schamel D., Salonen A., Drenckhan W., Gilchrist M.D., Stubenrauch C. (2012). Synthesis of Macroporous Polystyrene by the Polymerization of Foamed Emulsions. Polym. Foam..

[31] Gokmen M.T., Dereli B., De Geest B.G., Du Prez F.E. (2013). Complexity from Simplicity: Unique Polymer Capsules, Rods, Monoliths, and Liquid Marbles Prepared via HIPE in Microfluidics. Part. Part. Syst. Charact..

[32] Susec M., Ligon S.C., Stampfl J., Liska R., Krajnc P. (2013). Hierarchically porous materials from layer-by-layer photopolymerization of high internal phase emulsions. Macromol. Rapid Commun..

[33] Johnson D.W., Sherborne C., Didsbury M.P., Pateman C., Cameron N.R., Claeyssens F. (2013). Macrostructuring of emulsion-templated porous polymers by 3D laser patterning. Adv. Mater..

[34] Mao D., Li T., Liu H., Li Z., Shao H., Li M. (2013). Preparation of macroporous polyHIPE foams via radiation-induced polymerization at room temperature. Colloid Polym. Sci..

[35] Kircher L., Theato P., Cameron N.R. (2013). Reactive thiol-ene emulsion-templated porous polymers incorporating penta fluorophenyl acrylate. Polymer.

[36] Kimmins S.D., Wyman P., Cameron N.R. (2014). Amine-functionalization of glycidyl methacrylate-containing emulsion-templated porous polymers and immobilization of proteinase K for biocatalysis. Polymer.

[37] Moglia R., Whitely M., Brooks M., Robinson J., Pishko M., Cosgriff-Hernandez E. (2014). Solvent-free fabrication of polyHIPE microspheres for controlled release of growth factors. Macromol. Rapid Commun..

[38] Johnson D.W., Langford C.R., Didsbury M.P., Lipp B., Przyborski S.A., Cameron N.R. (2015). Fully biodegradable and biocompatible emulsion templated polymer scaffolds by thiol-acrylate polymerization of polycaprolactone macromonomers. Polym. Chem..

[39] Susec M., Liska R., Russmuller G., Kotek J., Krajnc P. (2015). Microcellular Open Porous Monoliths for Cell Growth by Thiol-Ene Polymerization of Low-Toxicity Monomers in High Internal Phase Emulsions a. Macromol. Biosci..

[40] Owen R., Sherborne C., Paterson T., Green N.H., Reilly G.C., Claeyssens F. (2016). Emulsion templated scaffolds with tunable mechanical properties for bone tissue engineering. J. Mech. Behav. Biomed. Mater..

[41] Whitely M., Rodriguez-River G., Waldron C., Mohiuddin S., Cereceres S., Sears N., Ray N., Cosgriff-Hernandez E. (2019). Porous PolyHIPE microspheres for protein delivery from an injectable bone graft. Acta Biomater..

[42] Langford C.R., Johnson D.W., Cameron N.R. (2014). Chemical functionalization of emulsion-templated porous polymers by thiol-ene “click” chemistry. Polym. Chem..

[43] Sušec M., Paljevac M., Kotek J., Krajnc P. (2016). Microcellular open porous polyester membranes from thiol-ene polymerisations of high internal phase emulsions. Des. Monomers Polym..

[44] Huš S., Kolar M., Krajnc P. (2016). Separation of heavy metals from water by functionalized glycidyl methacrylate poly (high internal phase emulsions). J. Chromatogr. A.

[45] Dikici B.A., Dikici S., Reilly G.C., Macneil S., Claeyssens F. (2019). A Novel Bilayer Polycaprolactone Membrane for Guided Bone Regeneration: Combining Electrospinning and Emulsion Templating. Materials.

[46] Hughes J.M., Budd P.M., Tiede K., Lewis J. (2014). Polymerized High Internal Phase Emulsion Monoliths for the Chromatographic Separation of Engineered Nanoparticles. J. Appl. Polym. Sci..

[47] Jiang Q., Barkan H., Menner A., Bismarck A. (2017). Micropatterned, macroporous polymer springs for capacitive energy harvesters. Polymer.

[48] Danninger D., Hartmann F., Paschinger W., Pruckner R., Schwödiauer R., Demchyshyn S., Kaltenbrunner M. (2020). Stretchable Polymerized High Internal Phase Emulsion Separators for High Performance Soft Batteries. Adv. Energy Mater..

[49] Jenjob R., Seidi F., Crespy D. (2018). Recent advances in polymerizations in dispersed media. Adv. Colloid Interface Sci..

[50] Debecker D.P., Boissiere C., Laurent G., Huet S., Eliaers P., Sanchez C., Backov R. (2015). First Acidic Macro-Mesocellular Aluminosilicate Monolithic foams ”SiAl(HIPE)” and their Catalytic Properties. Chem. Commun..

[51] Pan J., Zeng J., Cao Q., Gao H., Gen Y., Peng Y., Dai X., Yan Y. (2016). Hierarchical macro and mesoporous foams synthesized by HIPEs template and interface grafted route for simultaneous removal of λ-cyhalothrin and copper ions. Chem. Eng. J..

[52] Brunauer S., Emmett P., Teller E. (1938). Adsorption of Gases in Multimolecular Layers. J. Amer. Chem. Soc..

[53] Decker C. (2005). In situ monitoring of ultrafast photopolymerizations by real-time infrared spectroscopy. Polym. News.

[54] Jancovicova V., Kindernay J., Jacubikova Z., Mrlláková I. (2007). Influence of Photoinitiator and Curing Conditions on Polymerization Kinetics and Gloss of UV-Cured Coatings. Chem. Pap..

[55] Macarie L., Ilia G. (2005). The influence of temperature and photoinitiator concentration on photoinitiated polymerization of diacrylate monomer. Cent. Eur. J. Chem..

[56] Drache M., Stehle M., Mätzig J., Brandl K., Jungbluth M., Namyslo J.C., Schmidt A., Beuermann S. (2019). Identification of β-scission products from free radical polymerizations of butyl acrylate at high temperature. Polym. Chem..

[57] Barbetta A., Cameron N.R. (2004). Morphology and Surface Area of Emulsion-Derived (PolyHIPE) Solid Foams Prepared with Oil-Phase Soluble Porogenic Solvents: Span 80 as Surfactant. Macromolecules.

[58] Swedish Transmission Research Institute–STRI (1992). Guide 92/1 Hydrophobicity Classification Guide.

[59] Adamson A.W., Gast A.P. (1997). Physical Chemistry of Surfaces.

[60] Hiemenz P.C., Rajagopalan R. (1997). Principles of Colloids and Surface Chemistry.

[61] Thomazinia D., Gelfusoa M.V., Corrêa Altafim R.A. (2008). Hydrophobicity Classification of Polymeric Materials Based on Fractal Dimension. Mater. Res..

[62] Yamamoto M., Nishikawa N., Mayama H., Nonomura Y., Yokojima S., Nakamura S., Uchida K. (2015). Theoretical explanation of the Lotus Effect: Superhydrophobic property changes by removal of nanostructures form the surface of a Lotus leaf. Langmuir.

[63] Speight J.G., Shekhawat D., Spivey J.J., Berry D.A. (2011). Fuels for Fuell Cells in Fuel Cells: Technologies for Fuel Proceesing.

[64] Zhang T., Guo Q. (2017). Continuous preparation of polyHIPE monoliths from ionomer-stabilized high internal phase emulsions (HIPEs) for efficient recovery of spilled oils. Chem. Eng. J..

