# Supplementary material for: Development of Photocurable Polyacrylate-Based PolyHIPEs and the Study of the Kinetics of Photopolymerization, and of Their Thermal, Mechanical and Hydrocarbon Absorption Properties
Source: Polymers (Basel). 2021 Oct 12;13(20):3497. doi: 10.3390/polym13203497 (PMC8538452; doi:10.3390/polym13203497)
Supplement: Supplementary file 1 [file polymers-13-03497-s001.zip › polymers-1410396-supplementary .pdf]

Supplementary material

Development of Photocurable Polyacrylate-based PolyHIPEs and the Study of the Kinetics of Photopolymerization, and of their Thermal, Mechanical and Hydrocarbon Absorption Properties

Jefferson A. Reinoza Dávila, Ricardo Acosta Ortiz *, Ramiro Guerrero Santos

Centro de Investigación en Química Aplicada, Blvd Enrique Reyna # 140, Saltillo, Coahuila, Mexico, 25294, Tel +528444389844 email: ricardo.acosta@ciqa.edu.mx

ORCID Ricardo Acosta Ortiz 0000-0003-4824-9625, ORCID Ramiro Guerrero Santos 0000-0001-8346-5636, ORCID Jefferson Reinoza Dávila 0000-0002-9810-980X

Figure S1. Emission spectrum of Blue wave dymax UV-Vis lamp

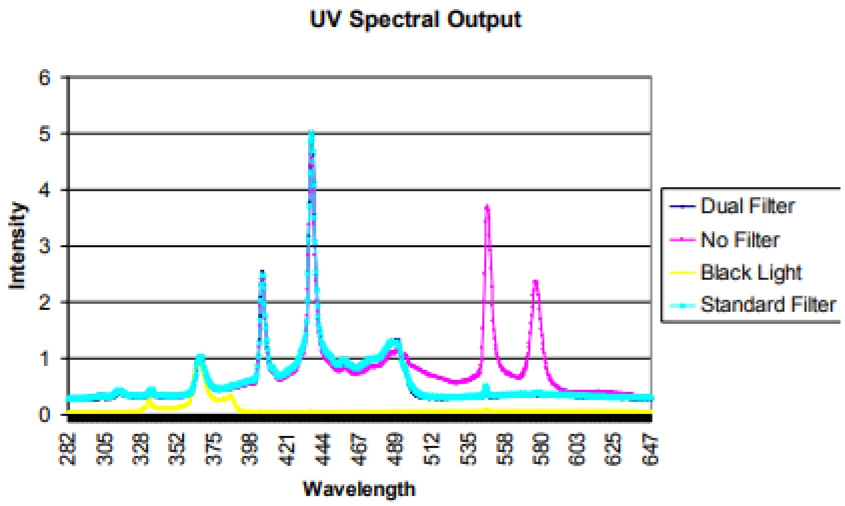

Emission spectrum of Blue wave 210 UV lamp taken from webpage https://www.intertronics.co.uk/product/bluewave-200-uv-curing-spot-lamp/

Figure S2a. Mechanism of photoinitiation of dimethoxyphenyl acetophenone (DMPA)

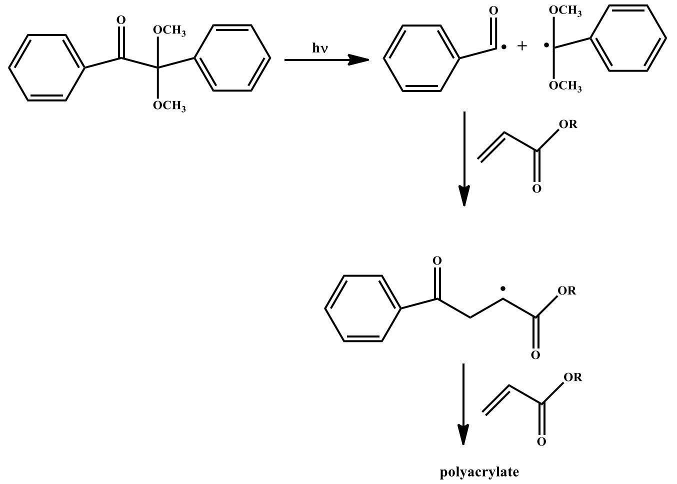

Figure S2b. Mechanism of generation of radical and initiation of photopolymerization of acrylates using BAPO

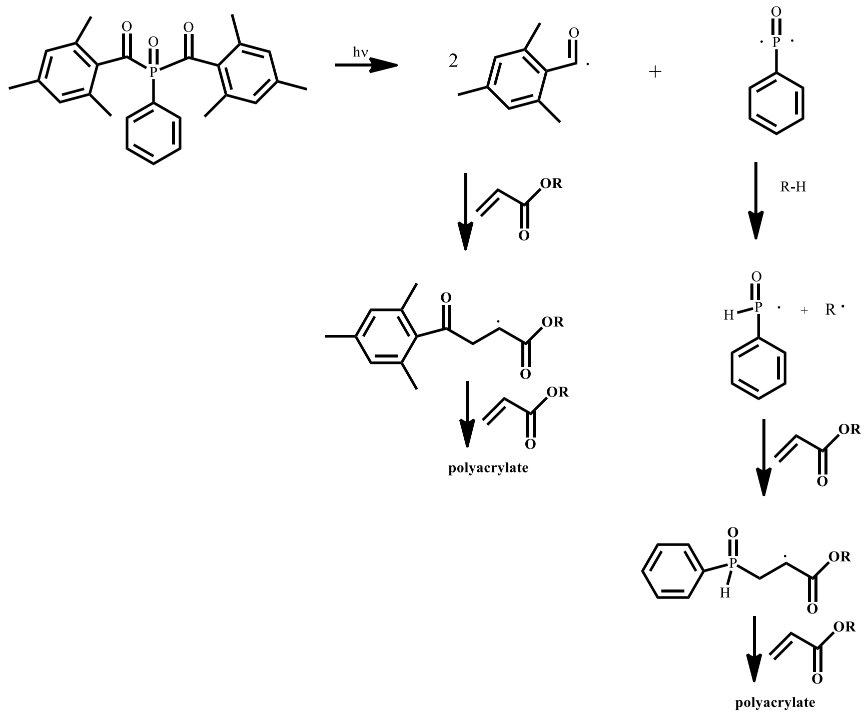

Video S1. Video of hydrophobic properties of polyHIPE derived from formulation T:E:I (1:0.9:2.1)
